# Orthosilicic acid inhibits human osteoclast differentiation and bone resorption

**DOI:** 10.1371/journal.pone.0312169

**Published:** 2024-10-15

**Authors:** Catarina Magnusson, Maria Ransjö

**Affiliations:** 1 Department of Orthodontics, Institute of Odontology, The Sahlgrenska Academy, University of Gothenburg, Gothenburg, Sweden; 2 Department of Odontology, Umeå University, Umeå, Sweden; University of Melbourne, AUSTRALIA

## Abstract

**Context:**

Silicon (Si), which is present in the diet in the bioavailable form of orthosilicic acid (OSA) and is detected as a dissolution product of certain bone-substitute materials, is suggested to promote bone health, and enhance bone healing, respectively. Silicon has been shown to stimulate osteoblastic cell differentiation and function, although the effect of Si on human osteoclasts is unclear.

**Aim:**

The present study investigated the direct effects of Si on human osteoclast differentiation, gene expression, and bone resorption.

**Material & methods:**

Human CD14+ monocytes were isolated from buffy coats and cultured with M-CSF and RANKL in medium without or with Si (50 μg/ml; constituting 75% OSA). The effects of Si on osteoclast differentiation were evaluated by TRAP-staining and the expression levels of *CtsK*, *CalcR*, *TRAP*, and *DC-STAMP* measured by RT-qPCR. The effect of Si on the expression level of *AQP9*, which encodes a potential Si transporter, was also analyzed. Bone resorption was determined based on the number of resorption pits formed when the RANKL-stimulated monocytes were cultured on bone slices, and by the levels of type I collagen fragments released into the cell culture medium.

**Results:**

Silicon significantly inhibited the number of TRAP+ multinucleated cells and the expression of osteoclast related genes but increased the late expression of *AQP9*. Furthermore, Si significantly inhibited the number of resorption pits and the amount of collagen fragments in the medium when cells were cultured on bone slices.

**Conclusion:**

Our results demonstrate that OSA inhibits RANKL-stimulated human osteoclast differentiation, gene expression of osteoclast phenotypic markers, and bone resorption.

## Introduction

The ubiquitous mineral silicon (Si) is suggested to be an important element for bone health [1]. The earliest reports on the physiological impact of Si involved deprivation studies conducted on chicks and rats, showing that Si deficiency inhibits normal skeletal development [2, 3]. Although the dramatic effects demonstrated in these reports have not been demonstrated since then, several observational and experimental studies have confirmed the positive effects of Si on bone metabolism. A comprehensive cross-sectional study carried out in the US found a positive association between the amount of Si in the diet and the bone mineral densities of subjects of both genders, although not in postmenopausal women [4]. Bone-promoting effects of Si supplementation have also been demonstrated in studies on ovariectomized rats, growing rats, and rats fed a Si-deficient diet [5–10].

Silicon is accessible to plants from the soil and ground-water in the monomeric form of orthosilicic acid (OSA) [Si(OH)_4_] [11]. However, the capacity to accumulate OSA differs widely among plants, which may be due to whether or not Si channels, through which OSA is transported into the plant xylem, are present in the roots [12]. Ma *et al*. were the first to describe a Si transporter, found in the roots of rice [12]. Suppression of the gene *Lsi1*, coding for a channel protein, reduced the accumulation of Si in the plants [12]. The transmembrane water channel proteins in animals and humans, called aquaporins (AQP), which are analogous to *Lsi1*, have the ability to transport Si, as demonstrated by transfection studies on *Xenopus laevis* oocytes and human HEK-293 cells. The human water channel protein aquaporin9 (AQP9) which facilitates transmembrane passage of water and small solutes, have also been proposed to permit the passage of OSA in mammalian cells [13]. Through the consumption of particularly plant-based food and beverages, humans assimilate Si in trace amounts. OSA is the only bioavailable form of Si, which means that larger Si species present in food need to be completely broken down to OSA for uptake in the intestine [14–17]. Most of the ingested Si is recovered in the urine and feces, although it is not clear as to whether any of the recovered Si is exchanged for the body-stored Si [18]. The levels of Si have not been examined in all human tissues, but Si has been found in trace amounts in human bone [19]. Studies aimed at comparing the Si levels in different tissues of animals have shown that connective tissues, including bone, contain comparatively high amounts of Si [20, 21].

Supra-physiological Si levels are suggested to be released locally in bone defects that have been grafted with Si-containing bone substitutes [22, 23]. Bio-Oss^®^, which is a bone-substitute material composed of deproteinized bovine bone, is used in alveolar bone augmentation, periodontal bone regeneration etc. [24]. Bio-Oss^®^ has been shown to release Si in cell culture media, albeit to varying degrees by different batches [22]. Bioactive glasses are synthetic alternatives used in dento-alveolar and cranio-facial bone reconstructions, e.g., with Bioglass 45S5 (PerioGlas^®^, NovaBone^®^), which contains a considerable amount of silica (~45% SiO_2_ by weight) [25]. *In vitro* studies have shown that the dissolution products (including Si) from bioactive glasses support osteoblast proliferation, phenotypic gene expression, and the production of extracellular matrix [26, 27]. Furthermore, studies investigating the effects of OSA at physiological concentrations have reported increased expression levels of osteogenic markers and enhanced osteogenic properties (alkaline phosphatase activity, collagen synthesis, nodule formation etc.) in mesenchymal cells [28–30].

We have earlier reported that ionic dissolution products from Bioglass 45S5 dose-dependently inhibit osteoclast differentiation, osteoclast-specific gene expression, and bone resorption in mouse bone marrow cultures [31]. Silicon, at the concentration released from bioactive glass, exerts significant inhibitory activities already in the early stages of osteoclast differentiation and reduces osteoclast bone-resorptive activity [31, 32]. However, the previous studies on the effects of Si on osteoclast differentiation and function were carried out on mouse cells either in co-cultures (i.e., bone marrow cultures) or with a monocyte/macrophage cell line (RAW264.7) [31–33]. To date, there has been only one report on the effect of Si on human osteoclasts, which has demonstrated a decreased number of differentiated cells and inhibition of bone resorption [30]. The aim of the present study was to investigate further the direct effects of Si on differentiation, gene expression, and bone resorption activities in primary human osteoclast precursors. The genes that were selected for expression analysis are well-known markers of osteoclast differentiation and function [34]. The genes encode the serine protease cathepsin K (CtsK), and tartrate-resistant acid phosphatase (TRAP), which both are secreted by the osteoclast during bone resorption are considered early markers for osteoclast differentiation [34]. Calcitonin receptor (CalcR) on the osteoclast cell membrane is considered as a late marker [34]. The dendritic cell specific transmembrane protein (DC-STAMP) that mediates cell-cell fusion during osteoclastogenesis is also considered as an important osteoclastic marker [35]. DC-STAMP is proposed to act by ligand-receptor interaction, but the ligand is still not identified. It has been suggested that DC-STAMP is a chemokine receptor and that the lack of cell fusion in *DC-STAMP*^*-/-*^ osteoclast precursors might be caused by impaired cell migration [35]. However, the fusion was abolished even when *DC-STAMP*^*-/-*^osteoclast precursors were cultured at high density and thus, cell-to-cell contact did not abrogate the inhibitory effect. The present theory today is that the DC-STAMP mediated fusion is dependent on ligands expressed on the precursors [35, 36].

To this end, we isolated human CD14+ monocytes from the peripheral blood and used cell culture media supplemented with Si at concentrations equivalent to the concentrations released from bioactive glass materials [31].

## Materials & methods

### Si-supplemented cell culture medium

The Si-supplemented cell culture medium was prepared as in the published protocols, and we used a non-toxic concentration within the range of Si concentrations released from bioactive glass [31, 32]. A stock solution of Si (350 μg/ml) was prepared by adding 100 μl of sodium silicate solution (reagent grade; Sigma-Aldrich Inc., St. Louis, MO, USA) to 49.9 ml of minimum essential medium (α-MEM; Gibco, Thermo Fisher Scientific Inc., Waltham, MA, USA) supplemented with 1% (v/v) antibiotic-antimycotic (Anti-Anti; Gibco) and 1% (v/v) L-alanyl-L-glutamine (200 mM GlutaMAX; Gibco). The pH of the Si stock solution was adjusted to 7.0−7.2 with HCl (37%; Sigma-Aldrich). The stock solution was supplemented with 10% (v/v) foetal bovine serum (FBS; Gibco), before further dilution with α-MEM. The final Si-supplemented medium (50 μg/ml; 1.8 mM) was left to stand for 24 hours prior to usage in cell culture. The concentration of Si (50 μg/ml) was verified in a previous report, together with an analysis revealing that 75% of the total Si content was OSA [32].

### Isolation of human CD14+ monocytes

For every isolation, fresh buffy coat was diluted (1:16) in phosphate-buffered saline solution (PBS; HyClone™; Cytiva, Boston, MA, USA) and centrifuged with Ficoll-Paque PLUS (Cytiva) for 20 minutes at 800 × *g* without interruption. In total, four buffy coats were obtained from the blood bank at Sahlgrenska University Hospital (Gothenburg, Sweden) from anonymous healthy donors. The peripheral blood mononuclear cell (PBMC) layer between the plasma and Ficoll was collected and washed in PBS three times and centrifuged for 10 minutes at 300 × *g*. CD14+ monocytes were isolated from the PBMCs by positive selection using CD14 MicroBeads (Miltenyi Biotec, Bergisch Gladbach, Germany) and magnetic separation using a MACS column (LS; Miltenyi Biotec) according to the manufacturer’s instructions. In brief, PBMCs were incubated with magnetic Microbeads conjugated with anti-human CD14 antibodies at 4°C for 15 minutes. The CD14+ monocytes were separated using a column placed in a magnetic field, which retained the beads with attached CD14+ cells, while the non-attached cells were allowed to flow through. The CD14+ monocytes were then collected from the beads by flushing the column outside the applied magnetic field.

### Cell culturing

CD14+ monocytes were incubated in α-MEM (including 1% Anti-Anti, 1% GlutaMAX and 10% FBS) that contained macrophage colony-stimulating factor (recombinant human M-CSF, 25 ng/ml; R&D Systems, Minneapolis, MN, USA) at 37°C in a humidified atmosphere with 5% CO_2_. Cells were seeded at a density of 500,000 cell/well in 24-well plates (Nunc; Thermo Fisher Scientific) for RNA isolation, and at 100,000 cells/well in 96-well plates for TRAP staining on plastic (Nunc; Thermo Fisher Scientific). Furthermore, 100,000 cells/well were seeded onto bone discs (Immunodiagnostic Systems Ltd., Boldon, UK), pre-incubated in α-MEM for at least 4 hours, and then placed in 96-well plates for bone resorption assays (toluidine staining and type I collagen release) or TRAP-staining. After at least 3 hours, non-adherent cells were removed, and the remaining adherent cells (either on plastic or on bone discs) were incubated in cell culture medium with or without Si supplementation (50 μg/ml). Receptor activator of nuclear factor κB (*E*. *coli*-expressed recombinant mouse RANKL; R&D Systems) was used in a concentration of 2 ng/ml for osteoclast differentiation on plastic and at 25 ng/ml on the bone discs. The culture medium was changed every second day. Cells were cultured on plastic for 48 h, 72 h, and 96 h for gene expression analyses and TRAP-staining. Cells cultured on bone were stained for TRAP after 7 days of culturing and bone resorption were analyzed between day 5 and 11 (toluidine staining and type I collagen release).

### Gene expression analyses

#### RNA isolation and reverse transcription

Total RNA samples were isolated after 48 h, 72 h, and 96 h of culturing, using the RNeasy Mini Kit (Qiagen, Hilden, Germany) according to the manufacturer’s protocol. RNA was eluted in 80 μl of RNase-free water and quantified with the Qubit RNA broad range assay kit using a Qubit fluorometer (Invitrogen, Thermo Fisher Scientific). RNA quality was assessed using a NanoDrop spectrophotometer (Thermo Fisher Scientific), and the RNA was kept at -80°C until cDNA synthesis. To control for inhibition of the synthesis of cDNA, the RNA was spiked with Universal RNA Spike II (TATAA Biocenter, Gothenburg, Sweden). Reverse transcription was carried out on a MiniOpticon (Bio-Rad Laboratories, Hercules, CA, USA) with the iScript gDNA Clear Synthesis kit (Bio-Rad Laboratories), including DNase treatment to eliminate genomic DNA.

#### qPCR

Gene expression analysis was performed on duplicates of each sample in a CFX Connect with the CFX manager software (Bio-Rad Laboratories). One ng of cDNA was added to each 10-μl reaction of SsoAdvanced Universal SYBR Green Supermix and predesigned primers (Table 1; Bio-Rad Laboratories). To enable comparisons of Cq-values between qPCR runs, an inter-plate calibrator (TATAA Biocenter) was added to each PCR-plate. The melting curve of each qPCR run was analyzed to check for inhibition due to primer hybridization. All target gene expression levels were normalized to the expression level of the validated reference gene Ubiquitin C (*UBC)*. The most-stable reference gene was determined by screening the expression patterns of twelve reference genes (TATAA Biocenter) with geNorm and NormFinder algorithms (GenEx software; MultiD Analyses AB, Gothenburg, Sweden).

**Table 1:** Bio-Rad SYBR^®^ Green primers.

Abbreviations	Gene symbol	Gene name	Unique assay ID
*CalcR*	CALCR	Calcitonin receptor	qHsaCID0022061
*CtsK*	CTSK	Cathepsin K	qHsaCID0016934
*TRAP*	ACP5	Acid phosphatase 5, tartrate-resistant	qHsaCED0056724
*DC-STAMP*	TM7SF4	Transmembrane 7 superfamily member 4	qHsaCID0021541
*AQP9*	AQP9	Aquaporin 9	qHsaCID0016607
*UBC*	UBC	Ubiquitin C	qHsaCED0023867

### Osteoclast formation assay

#### Fixation and TRAP-staining

Cells that were cultured on plastic plates were washed twice with PBS and fixed with citrate-acetone (1:4 dilution of 27 mM citrate solution in ≥99.5% acetone) for 30 seconds. Cells that were cultured on bone slices were washed twice with PBS and fixed with formaldehyde solution (4%; Histolab AB, Askim, Sweden) for 15 minutes at room temperature. The cells were washed thoroughly with deionised water after fixation. Cells were stained for TRAP using the Acid Phosphatase Leukocyte staining kit (Sigma-Aldrich) according to the manufacturer’s instructions, except for a reduced staining time of 20 minutes for plastic and 40 minutes for bone substrate. Cells fixed on plastic were counted under a light microscope by a single observer and classified as osteoclasts if they stained positively for TRAP (dark red) and had three or more nuclei.

### Bone resorption assays

#### Toluidine staining

For assaying bone resorption, cells were detached from the bone slices by trypsinization (Trypsin-EDTA 0.25%; Thermo Fisher Scientific) and rubbing with cotton swabs. Bone slices were cleaned of cell debris by repeated washing with PBS. Resorption pits were stained with freshly prepared toluidine blue (0.1% w/v in Milli-Q water, filtered) for 15 minutes at room temperature. The stained bone slices were washed thoroughly with PBS. A confined blue area on the bone, appearing either circular or multilobulated, was counted one resorption pit. The numbers of resorption pits were counted under a light microscope by a single observer.

#### CTX-I ELISA

The amount of released of c-terminal type I collagen (CTX-I) fragments in the cell culture medium during bone resorption was assayed by ELISA using a commercially available kit (CrossLaps for culture; Immunodiagnostic Systems). The supernatants were collected at every medium change and the ELISA was performed according to the manufacturer’s instructions. In brief, supernatants containing CTX-I and standards were pipetted into streptavidin-coated wells. A mixture of an antigen-specific antibody (biotinylated) and an enzyme-conjugated (horseradish-peroxidase, HRP) antibody was added to the wells. The wells were washed thoroughly, tetramethylbenzidine (TMB) was added as substrate for the HRP to yield a color, and H_2_SO_4_ was added to stop the color reaction. The levels of CTX-I in the supernatants were quantified by measuring the absorbance of the color at 450 nm on a microtiter plate reader (Multiskan FC; Thermo Fisher Scientific) followed by interpolation to the obtained standard curve constructed with known concentrations.

### Statistical analyses

Statistical analyses of the data and related graphs were made with the GraphPad Prism 9 software (GraphPad Software Inc., San Diego, CA, USA). The Shapiro-Wilk test was used to evaluate whether or not the data were normally distributed. To determine whether there was a statistical difference between cells cultured with or without Si, an unpaired *t*-test was used for each time-point in the gene expression assay, in the resorption assay for the number of pits, and in the resorption assay for the amount of CTX-I released. Statistics on the gene expression data were calculated from the ΔC_q_ values (Cq(gene of interest)—C_q(UBC)_) and presented as fold change calculated according to the 2^-ΔΔCq^ method [37]. Data and summary statistics for each figure can be found in S1 Data. A significance level of 0.05 was used for all the tests performed.

### Ethics statement

Buffy coats were provided by the blood bank at Sahlgrenska University Hospital (Gothenburg, Sweden). There was no requirement for ethical approval for the use of buffy coats as they were received in de-identified form and the donors had accepted use in scientific purpose.

## Results

### Effect of Si on osteoclast differentiation

**Fig 1:**
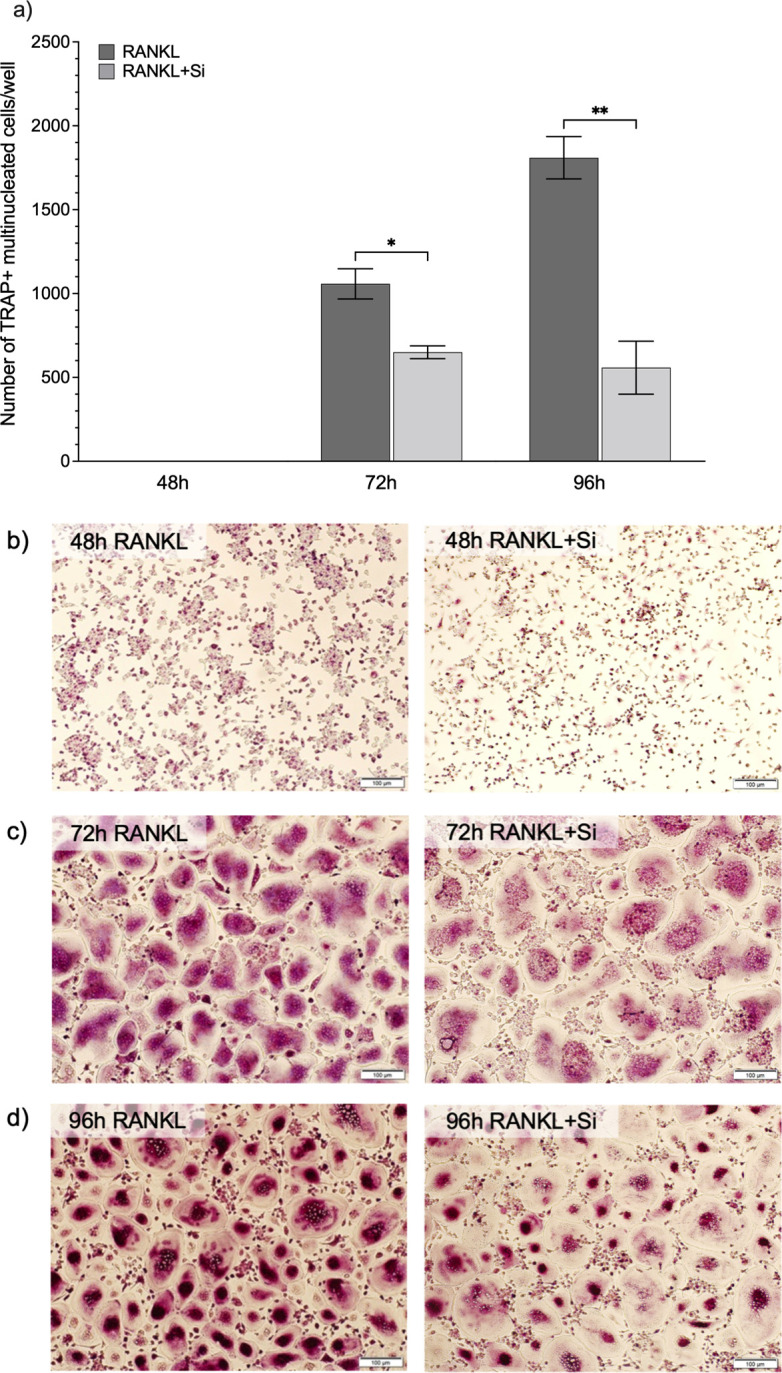
Effects of silicon on human osteoclast differentiation. CD14+ monocytes were stimulated with M-CSF (25 ng/ml) and RANKL (2 ng/ml), and cultured in either medium alone (RANKL) or in Si-supplemented medium (50 μg/ml; RANKL+Si). The numbers of TRAP+ multinucleated cells (mean ± SEM) of 3 samples (n = 3) detected after 48 h, 72 h and 96 h in culture are shown in (a), together with representative images of the respective cells after 48 h (b), 72 h (c), and 96 h (d) (scale bar equals 100 μm). Statistically significant differences between cells cultured without or with Si were tested with an unpaired *t*-test; *p <0.05, and ** *p* <0.01.

No TRAP+ multinucleated cells were observed after 48 h in the RANKL-stimulated CD14+ monocyte cultures, either in the absence or presence of Si (Fig 1). There were, however, high numbers of TRAP+ multinucleated cells after 72 h (1,058 ± 90, mean ± SD) and after 96 h (1,809 ± 126) of RANKL stimulation (2 ng/ml). The presence of Si (50 μg/ml) significantly reduced the numbers of TRAP+ multinucleated cells, both at 72 h (650 ± 38, *p* = 0.014) and 96 h (558 ± 158, *p* = 0.04).

### Effect of Si on osteoclastic gene expression

**Fig 2:**
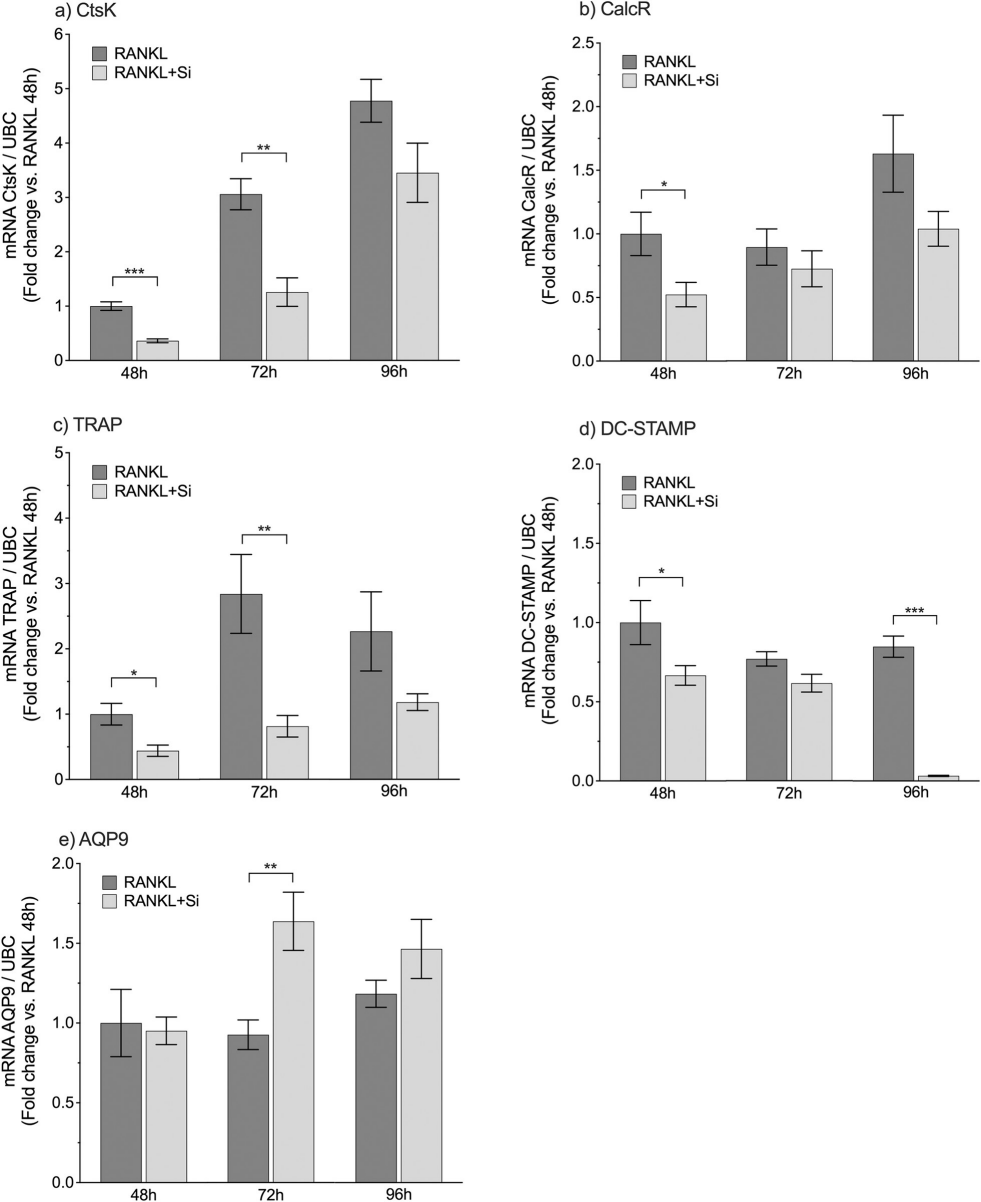
Effects of silicon on the gene expression levels in human monocytes. Effects of Si (50 μg/ml) on the expression levels of *CtsK*, *CalcR*, *TRAP*, *DC-STAMP*, and *AQP9* in RANKL-stimulated (2 ng/ml) CD14+ monocytes, analyzed after 48 h, 72 h, and 96 h in culture. Gene expressions are normalized to the reference gene *UBC* and are presented as mean fold change relative to RANKL 48 h (RANKL 48 h set to 1) ± SEM of five to six samples of one representative experiment (n = 5–6). Differences between cells cultured in medium alone (RANKL, dark gray) and in Si-supplemented medium (RANKL+Si, light gray) were tested with an unpaired *t*-test at each time-point and statistical significance is indicated by: *p <0.05, **p <0.01, and ***p <0.001.

The expression levels of *CtsK*, *CalcR*, *TRAP*, *DC-STAMP*, and *AQP9* were analyzed at 48 h, 72 h, and 96 h in the RANKL-stimulated (2 ng/ml) CD14+ monocytes cultured with or without Si (50 μg/ml) (Fig 2). The expression levels of *CtsK* and *TRAP* were significantly decreased by Si at 48 h (*p* < 0.001, *p* = 0.022, respectively) and 72 h (*p* = 0.003, *p* = 0.005, respectively), as compared to untreated cells. Significant inhibition of *CalcR* expression by Si was only detected at 48 h (*p* = 0.045), whereas *DC-STAMP* expression was inhibited at 48 h (*p* = 0.049) and 96 h (*p* < 0.001). In contrast to the other genes, the expression of *AQP9* was increased by Si, significantly however only at 72 h (*p* = 0.004).

### Effect of Si on bone resorption

**Fig 3:**
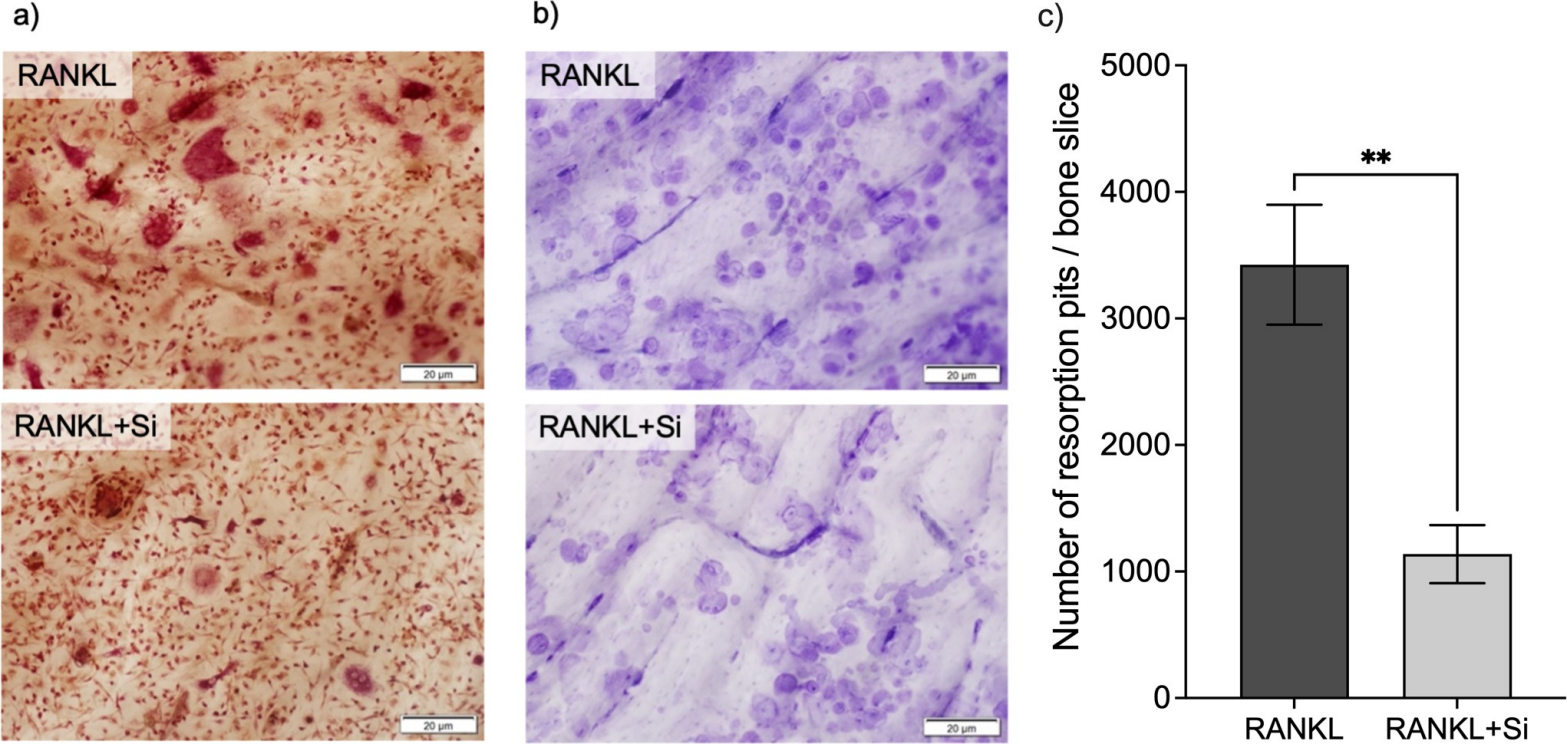
Effects of silicon on human osteoclast differentiation and bone resorption. CD14+ monocytes were cultured on bone slices and stimulated with M-CSF (25 ng/ml) and RANKL (25 ng/ml), and cultured in medium alone (RANKL) or in Si-supplemented medium (50 μg/ml; RANKL+Si). The effect of Si on the number of osteoclasts is shown in representative images of TRAP-stained cells on Day 7 (scale bar equals 20 μm) (a). The effects of Si on the numbers of resorption pits on Day 11 are shown in representative images of toluidine-stained bone discs (b) and presented as mean ± SEM of five discs (n = 5) in panel (c). Statistically significant differences between the groups were tested with an unpaired *t*-test; **p <0.01.

Monocytes cultured on bone discs in the presence of RANKL (25 ng/ml) for 11 days produced a high number of resorption pits (3,425 ± 1,060, mean ± SD; Fig 3). Significantly fewer resorption pits (1,138 ± 513; *p* < 0.003) were counted on bone discs in RANKL-stimulated cultures with Si-supplemented medium (50 μg/ml).

**Fig 4:**
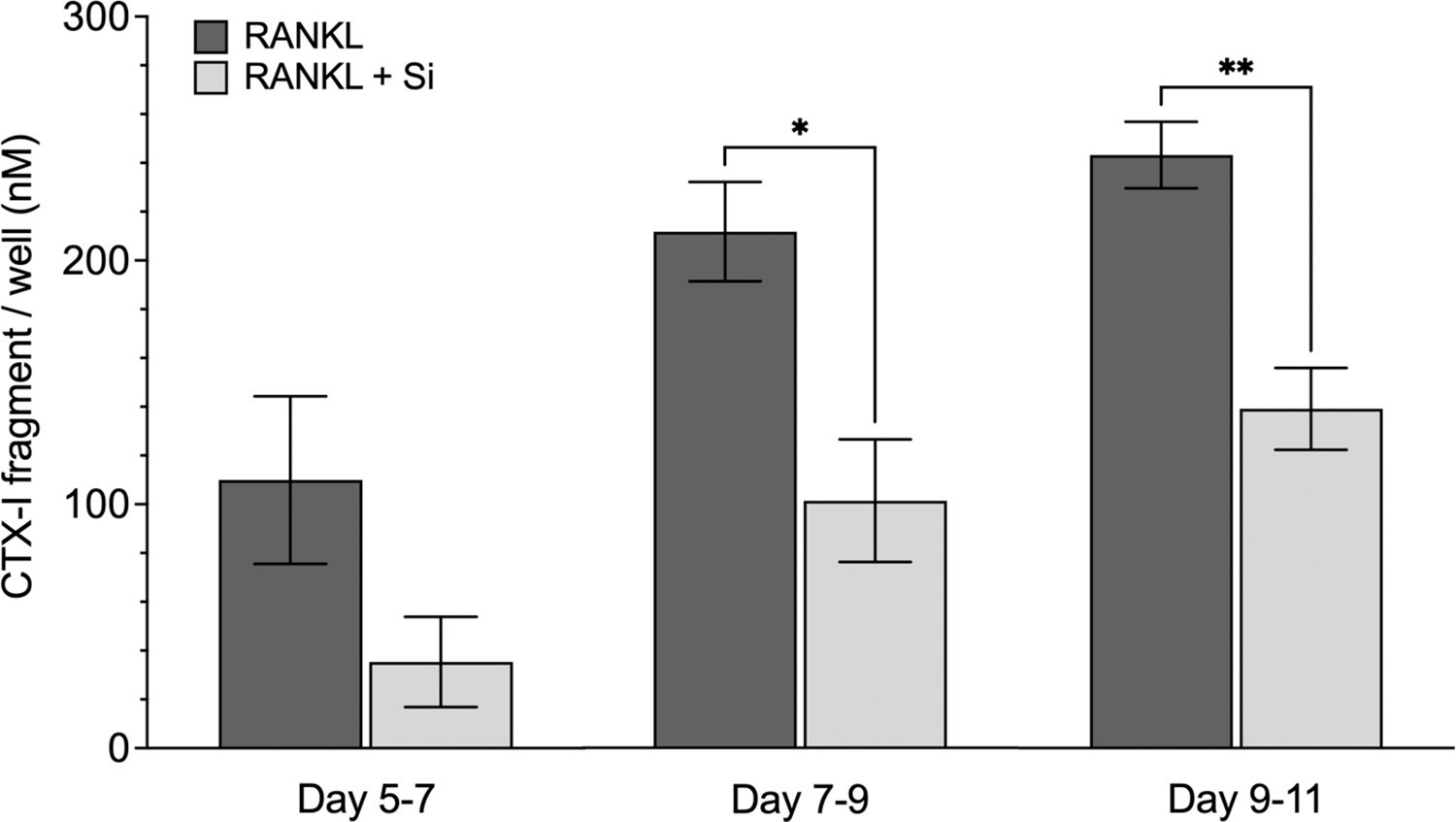
Effect of silicon on osteoclastic bone resorption, assayed as released CTX-I. CD14+ monocytes were cultured on bone discs and stimulated with M-CSF (25 ng/ml) and RANKL (25 ng/ml), and cultured in either medium alone (RANKL, dark gray) or in Si-supplemented medium (50 μg/ml; RANKL+Si, light gray). The amounts of released type-I collagen fragments (CTX-I, nM) from the resorption process at different time-points are presented as mean ± SEM of three to four samples (n = 3–4). Statistically significant differences between the groups were tested with an unpaired *t*-test; *p <0.05, and ** *p* <0.01.

The amounts of CTX-I in the supernatants obtained from RANKL-stimulated (25 ng/ml) monocytes cultured in the absence or presence of Si (50 μg/ml) were 110 nM (SD; ± 69) and 35 nM (± 37) on Days 5–7, 213 nM (± 35) and 102 nM (± 44) on Days 7–9, 244 nM (± 27), and 140 nM (± 34) on Days 9–11, respectively (Fig 4). There was a significant reduction in the amount of CTX-I released in the RANKL-stimulated cultures with Si compared to those without Si on Days 7–9 (*p* < 0.027), and Days 9–11 (*p* < 0.003).

## Discussion

In addition to evaluating the effect of Si on the number of osteoclasts, we examined the effect of Si on the gene expression of five markers with relevance to osteoclast differentiation (*DC-STAMP*), the osteoclast phenotype (*CtsK*, *CalcR*, *TRAP*), and the proposed transport of Si across the plasma membrane (*AQP9*).

After 48 h of RANKL stimulation, no clear difference was observed between the cultures with or without Si that both contained only mononuclear cells, some of which were clustered together. However, the expression levels of the osteoclast phenotypic genes *CtsK*, *CalcR*, and *TRAP* were all significantly lower at 48 h in Si treated cells, which is in line with our previous reported inhibitory effect of Si on gene expression in RAW264.7 cells [32]. Compared to 48 h, there were significantly higher expression levels of *CtsK* and *TRAP* in the RANKL-stimulated cells at 72 h (S2 Data; *p* <0.001 and *p* <0.01, respectively), albeit still inhibited by Si.

Moreover, our present study demonstrates that Si reduced the gene expression of *DC-STAMP*, significantly at 48 h and at 96 h. The inhibition of the DC-STAMP-dependent fusion of the preosteoclastic monocytes is confirmed by the fewer multinucleated cells detected in the RANKL-stimulated cultures treated with Si. The expression of *DC-STAMP* has been found previously to be inhibited by Si in RANKL-stimulated RAW264.7 cells and mouse bone marrow macrophages at late differentiation [32, 38].

Several AQPs have been suggested to be involved in the transmembrane transport of Si [13], but only *AQP9* expression has been reported in osteoclasts (murine bone marrow macrophages and RAW264.7 cells) [39]. The present study is the first showing the expression of *AQP9* in human osteoclasts. Results from the gene expression assays suggests that Si increases the expression of *AQP9*, significant at 72 h. This supports the paper by Garneau *et al*. describing significantly higher expression of *AQP9* in the calvarial bone of mice fed a Si-rich diet compared to those fed a Si-low diet [13]. Possible explanations for the increased expression of *AQP9* by Si can only be speculated about. If AQP9 is a transporter of Si during the differentiation of preosteoclasts to osteoclasts, the increased expression of *AQP9* in Si-exposed cells might then be a direct consequence of the abundance of Si in the vicinity of the cell, i.e., the gene is up-regulated to allow more Si to traverse the cell membrane aided by AQP9. However, in a paper investigating the role of AQP9 on murine osteoclastogenesis, it is claimed that AQP9 is essential for the cytosol expansion by influx of water, during the fusion step of osteoclastogenesis [39]. Since our results indicates that Si enhances the *AQP9*-expression but also inhibits osteoclastogenesis, the results of that paper [39] are not consistent with ours. A limitation of the present study is that no blocking assay was performed. Phloretin has been demonstrated to inhibit osteoclastogenesis by blocking AQP9 [39] but since phloretin is unspecific, it cannot be used for evaluating the possible AQP9 Si transport. However, the involvement of AQP9 in bone cell Si transport needs to be investigated further, ideally by a specific pharmacological blocker or by genetic editing of *AQP9*.

Inhibition of bone resorption by soluble Si has previously been demonstrated in RANKL-stimulated CD14+ cells cultured on synthetic calcium phosphate films [30]. Furthermore, Si significantly has been found to inhibit bone resorption in RANKL-stimulated mouse calvarial bone cultures [31]. Based on the reduced number of resorption pits on the bone discs and the reduced concentration of released CTX-I in the culture medium, as demonstrated in the present study, it can be concluded that Si inhibits osteoclastic bone resorption. However, since Si inhibits the number of differentiated osteoclasts, the reduced bone resorption observed with Si could be a consequence of fewer mature osteoclasts rather than a blocking of the resorptive capacity per se of the individual cells. Nonetheless, a therapeutic agent that inhibits the differentiation of osteoclasts and, thereby, inhibits bone resorption is also clinically relevant [40]. This study confirms the favorable features of Si. Si does not only stimulate osteogenesis, but also inhibit osteoclast differentiation and function, thus promoting bone health in a synergistic manner. This may be of interest in the development of new generations of bone graft materials and provide clues into the possible importance of dietary Si. Furthermore, the results of the gene expression analyses confirms that *AQP9* is expressed in osteoclasts and regulated by Si, thus making AQP9 interesting to further investigate as a transporter of Si in mammals.

In conclusion, our results demonstrate that OSA inhibits RANKL-stimulated human osteoclast differentiation, gene expression of osteoclast phenotypic markers, and bone resorption.

## Supporting information

S1 Data Excel spreadsheet containing, in separate tabs, the numerical data and statistics underlying Figs 1A, 2A-2E, 3C and 4. (XLSX)

S2 Data Excel spreadsheet containing, in separate tabs, numerical data and statistics for comparison of the expression of Ctsk and TRAP between 48 h and 72 h in RANKL-stimulated cells. (XLSX)
